# Substance use and treatment utilization patterns of working-age American men who were not in employment, education, or training (NEET) during the COVID-19 pandemic

**DOI:** 10.1186/s13011-026-00733-6

**Published:** 2026-05-23

**Authors:** Wayne Kepner, Keith Humphreys

**Affiliations:** 1 Department of Psychiatry and Behavioral Sciences, Stanford University School of Medicine, 1070 Arastradero Rd Stanford, Palo Alto, CA 94305 USA; 2 Center for Innovation to Implementation, Veterans Affairs Health Care System, Palo Alto, CA USA

**Keywords:** COVID-19, Epidemiology, NEET, Opioid use, Substance use disorder, Substance use treatment, Unemployment

## Abstract

**Background:**

A growing population of working-aged men are not in employment, education, or training (NEET). The COVID-19 pandemic has been associated with changes in rates of substance use disorders (SUDs) and treatment seeking in the general population, but COVID-era substance use patterns among NEET men are unknown.

**Methods:**

We estimated the prevalence and correlates of NEET status among working-aged (18–64) men using data from the 2022 National Survey on Drug Use and Health, a nationally representative survey of non-institutionalized individuals in the United States. We developed logistic regression models to examine associations between NEET status and substance use behaviors and treatment engagement, adjusted for sociodemographic factors.

**Results:**

An estimated 11.1% of working-aged men were NEET in 2022 representing 10.6 million individuals. Compared to non-NEET men, NEET men were significantly overrepresented among older age groups, lower income brackets, unmarried individuals, those with lower educational attainment, and Non-Hispanic Black men. After adjusting for sociodemographic factors, NEET status was significantly associated with higher odds of prescription tranquilizer/sedative use disorder (aOR = 3.54, 95% CI: 1.97–6.37), methamphetamine use disorder (aOR = 3.10, 95% CI: 1.82–5.28), and prescription pain‑reliever use disorder (aOR = 2.88, 95% CI: 1.82–4.53), while being inversely associated with alcohol use disorder (aOR = 0.68, 95% CI: 0.54–0.85).

**Conclusion:**

More than 1 in 10 working-aged men were NEET in 2022. Adjusted models showed higher odds of past-year SUDs but lower odds of alcohol use disorder. Targeted interventions should include age-appropriate, culturally tailored, and substance-specific treatment programs to improve public health.

## Introduction

The decline in American male workforce participation has created a growing population of men who are “not in employment, education or training” (i.e., NEET); estimates suggest that 11% of prime-aged men (aged 25–54) were not participating in the labor force in 2019 [1, 2]. According to the Bureau of Labor Statistics (BLS), individuals are classified as “unemployed” if they are jobless, actively seeking work, and available for employment [3]. Those who are neither employed nor actively seeking work are classified as “not in the labor force” and are excluded from the official unemployment rate. This group includes discouraged workers, caregivers, those in school and individuals with disabilities. As a result, traditional metrics of unemployment typically capture active jobseekers; they exclude individuals who are long-term unemployed, not looking for work, and at risk of permanent workforce exclusion. The NEET concept provides a broader framework by capturing both the unemployed and those not in the labor force who are also not engaged in education or training.

The COVID-19 pandemic created disruptions to both employment and substance use patterns among American men. Economic downturns differently affect men’s substance use behaviors compared to women, and men’s overrepresentation in recession-vulnerable sectors like manufacturing and construction may further widen existing gender disparities in substance use behaviors [4, 5]. Similar patterns emerged during the Great Recession, when job losses among men were tied to rising “deaths of despair” [6, 7]. During the pandemic, labor force participation rates among prime-aged men (25–54) dropped to 86.4% (in April of 2020), meaning that 13.6% were not in the labor force [1]. COVID-19 and measures associated with it (e.g., social distancing measures, business closures) exerted substantial effects both on substance use patterns and treatment seeking for substance use disorders (SUD) [8]. These disruptions may reflect complex, potentially reinforcing cycles, as SUD may perpetuate NEET status [8]. Employment upheavals also varied by industry, which led to significant disparities among vulnerable socio-economic populations who were disproportionately impacted by pandemic-related job losses and substance-related harms [9, 10]. Therefore, there is an urgent public health need to better understand the demographic profile, substance use behaviors, and SUD treatment utilization patterns of NEET men during the COVID-19 pandemic [11].

Multiple studies have identified bi-directional links between substance use disorders and NEET status among men. In a national longitudinal cohort, Manhica et al. demonstrated that NEET status among young adults (ages 17–24) was associated with an increased risk of later SUD diagnosis among males 25 and older [12, 13]. In a study of 4,758 young Swiss men in their early 20s, previous cannabis use, and daily tobacco smoking predicted an increased likelihood of being NEET over 15 months [14]. Rodwell demonstrated that adolescents (14–15) who used cannabis frequently (defined as at least weekly use) had a 74% higher likelihood of being NEET in young adulthood (24–25) compared to those who used cannabis infrequently or not at all. Although Rodwell’s study included both males and females, males had higher rates of frequent cannabis use during adolescence (19.3% of males versus 11.9% of females). Finally, Compton et al. showed that unemployed men and women aged 18 and older (defined as without a job in the past four weeks) had 40% higher prevalence of heavy alcohol use and 79% higher prevalence of illicit drug use than employed individuals [15]. However, existing research on NEET men has typically focused on young adults and frequently lacked diverse samples, limiting generalizability to the broader population of working-age men and to the unique circumstances of the COVID-19 pandemic.

Pandemic-related social and economic disruptions reshaped both substance-use behaviors and pathways to treatment [8]. The US experienced an average increase in alcohol use [16], drug overdose rates and SUD hospitalizations [17, 18], and a decrease in tobacco use [19], with mixed evidence of increases in cannabis use [20]. The COVID-19 pandemic created barriers to SUD treatment seeking through social distancing measures, but the relaxation of regulations on telehealth and medications for opioid use disorder may have helped counter forces that reduced treatment access [21, 22]. Evidence on changes in health services access for SUD are varied and fragmented across different study settings [23]. Moreover, these changes may have had disproportionate impacts among vulnerable populations such as NEET men.

To help fill these knowledge gaps, we examined sociodemographic factors, substance use patterns, and SUD treatment utilization among working-age male participants in the 2022 National Survey on Drug Use and Health (NSDUH). Characterizing these patterns using national data from this timeframe is essential for developing targeted interventions since changes in substance use patterns may signal increased need for specific treatment strategies and workforce development programs. Our primary objectives are to: (1) characterize substance-specific use patterns among working-age NEET vs. non-NEET men, (2) examine their patterns of SUD treatment utilization, and (3) identify specific demographic variables for potential targeted interventions. These data can help develop policies and programs designed to help NEET men re-enter the workforce or education, mitigate the negative SUD-related consequences of NEET status, and increase national preparedness for future labor shocks [24–26].

## Methods

### Data source

We analyzed data from the 2022 National Survey on Drug Use and Health (NSDUH), an annual cross-sectional federal government survey of non-institutionalized individuals in the United States. We selected the 2022 NSDUH because it was the most recent publicly available data at the time of analysis that captured a period when both pandemic-related disruptions to employment and substance use treatment policies were still in effect while survey methodology had stabilized. The 2020 and 2021 NSDUH had significant methodological changes due to the pandemic including a transition to web-based data collection [27]. The NSDUH obtains a nationally representative probability sample through a complex survey design for all 50 states and the District of Columbia [28]. Surveys are administered via computer-assisted interviewing and audio computer-assisted self-interviewing. The analytic sample included 27,047 men of working age (18–64 years) from the 2022 NSDUH dataset. The 2022 NSDUH had a screening response rate of 25.46% and a weighted interview response rate of 47.43%. To address potential nonresponse bias, the survey implements demographic-based weight adjustments, comprehensive nonresponse bias analyses, and targeted follow-up with nonrespondents [28, 29]. The study used publicly available and de-identified data and was exempt from IRB review.

### Measures

The primary outcome was NEET status, defined as : (1) not working in the past year, (2) not currently enrolled in school or training programs, and (3) not retired. NEET status was operationally defined using three NSDUH items: whether the respondent worked at any job in the past 12 months, whether they were currently enrolled in school or training, and their work situation in the past week (used to identify and exclude retirees). This definition captures longer-term disengagement from work and education rather than short-term unemployment. NEET status was coded as a binary variable. We defined “working age” as 18–64 years rather than the stricter “prime-age” definition (25–54) because the NEET concept explicitly includes individuals who are neither in education nor training. Excluding 18–24 year-olds would have omitted a critical population of young men who could reasonably be expected to be enrolled in post-secondary education or vocational training. The upper limit of 64 aligns with standard pre-retirement working-age definitions. Cases with missing data on employment, education, or retirement status were excluded from analyses. All other variables had complete data due to NSDUH’s imputation procedures. We considered the following demographic characteristics: age (18–25, 26–34, 35–49, and 50–64 years), race/ethnicity (Non-Hispanic (NH) Asian, NH Black/African-American, Hispanic, NH Native American/Alaskan Native, NH Native Hawaiian/Pacific Islander, NH Multiracial, and NH White), education (less than high school, high school graduate, some college, and college graduate), annual family income (<$20,000, $20,000-$49,999, $50,000-$74,999, and >$75,000), relationship status (never married, married, and unmarried with the latter including widowed, divorced and separated individuals), and urbanicity (large metropolitan, small metropolitan, and non-metropolitan).

Past-month substance use was assessed for each substance through self-report.

The specific substances included alcohol, ecstasy/molly, LSD, cannabis, cocaine, heroin, fentanyl, methamphetamine, prescription opioids, prescription stimulants, prescription pain relievers and tranquilizers/sedatives. Misuse was defined as use of prescription drugs in any way a doctor did not direct [29]. We assessed past-month nicotine dependence defined by NSDUH using the Nicotine Dependence Syndrome Scale [30]. We further assessed substance use frequencies of cannabis, heroin, methamphetamine and alcohol. SUD outcomes included past-year disorders for alcohol and/or drugs, as defined by DSM-5 criteria derived from NSDUH survey items [31]. Specific SUD outcomes examined included alcohol use disorder, cannabis use disorder, cocaine use disorder, methamphetamine use disorder, prescription opioid use disorder, heroin use disorder, any opioid use disorder (heroin or prescription opioid), prescription stimulant use disorder, prescription tranquilizer/sedative use disorder, and any substance use disorder (drug or alcohol use disorder). Substance use treatment was categorized as a binary variable that included receiving treatment for alcohol or drug use at any location, such as a hospital, rehabilitation facility, mental health center, emergency room, or doctor’s office, including phone or video treatment.

### Statistical analysis

We estimated the prevalence of NEET status among working-age men and compared demographic characteristics, substance use behaviors, and SUD treatment utilization patterns between NEET and non-NEET men. Bivariable comparisons were conducted using Rao-Scott chi-square to determine differences for each of these conditions with respect to NEET status. To examine the association between NEET status and our outcomes of interest, we developed logistic regression models and adjusted for selected demographic characteristics (age, race/ethnicity, marital status, and urbanicity).

To account for potential Type 1 error resulting from multiple comparisons, we used familywise Bonferroni statistical correction for the 7 demographic variables (α = 0.007; 0.05/7) and the 11 outcomes variables (α = 0.0045; 0.05/11) to determine significance. We assessed multicollinearity among covariates using variance inflation factors (VIF); all VIFs were below 5 which indicates no problematic multicollinearity. Weights were included in all analyses and R Studio version 4.3.2 was used to analyze data [32]. The analysis incorporated NSDUH’s complex survey design using cluster weights, strata weights, and calibrated person-level weights [33].

## Results

### Sample characteristics

The largest proportions of respondents were heterosexual (88.9%, 95% CI: 87.9–89.8), non-Hispanic White (58.6%, 95% CI: 56.7–61.0), aged 35–49 (31.5%, 95% CI: 30.5–32.5) or 50–64 (30.6%, 95% CI: 29.1–32.2), married, (45.1% CI: 43.6–46.6), and resided in large metropolitan areas (55.6%, 95% CI: 53.3–57.9). An estimated 11.1% (95% CI: 10.1–12.1) of working aged (18–64) males in the US were not in employment, education or training (i.e., NEET) representing an estimated 10.6 million individuals (95% CI: 9.7–11.6 million). Table 1 presents demographic characteristics according to NEET status. Significant racial/ethnic disparities were observed with Non-Hispanic Black men showing higher NEET rates (18.5% vs. 11.5%) and Non-Hispanic Asian men showing lower rates (1.8% vs. 6.9%). Individuals reporting gay sexual orientation had significantly higher NEET rates compared to individuals reporting heterosexual orientation (7.5% vs. 3.7%). Compared to non-NEET men, NEET men were significantly more likely to be older (46.6% aged 50–64 vs. 28.7%), have lower income (45.9% earning <$20,000 vs. 10.4%), and less likely to be married (29.5% vs. 47.3%). They were also less likely to be college graduates (11.0% vs. 34.0%) and more likely to live in non-metropolitan areas (17.6% vs. 11.4%). All demographic differences were statistically significant (ps < 0.007).

**Table 1:** Demographic Characteristics of Working-Aged Men (18-64y) by NEET Status, United States, 2022 National Survey on Drug Use and Health

Characteristic	Full Sample ages 18–64 weighted % (95% CI)	Not NEET ages 18–64 weighted % (95% CI)	NEET ages 18–64 weighted % (95% CI)	*p*-value
**Sexual orientation**				
Heterosexual	88.9 (87.9–89.8)	91.3 (90.3–92.2)	86.1 (83.3–88.6)	< 0.001
Gay	4.1 (3.5–4.7)	3.7 (3.1–4.4)	7.5 (5.3–10.4)	
Bisexual	3.8 (3.3–4.3)	3.8 (3.3–4.4)	4.5 (3.2–6.4)	
**Race/ethnicity**				
NH Asian	6.2 (5.4–7.2)	6.9 (5.9–7.9)	1.8 (1.1–2.9)	< 0.001
NH Black / African American	12.2 (11.4–13.1)	11.5 (10.6–12.4)	18.5 (15.6–21.8)	
Hispanic	19.9 (18.2–21.9)	19.7 (17.7–21.8)	18.9 (15.2–23.2)	
NH Native American / Alaskan Native	0.5 (0.4–0.7)	0.5 (0.4–0.7)	0.6 (0.3–1.2)	
NH Native Hawaiian / Pacific Islander	0.6 (0.4-1.0)	0.6 (0.4-1.0)	0.4 (0.1–1.4)	
NH Multiracial	1.9 (1.5–2.2)	1.8 (1.5–2.2)	2.1 (1.4–3.2)	
NH White	58.6 (56.7–60.5)	59.1 (57.1–61.0)	57.7 (52.9–62.4)	
**Age**				< 0.001
18–25, years	17.6 (16.7–18.5)	18.4 (17.5–19.5)	11.8 (10.2–13.6)	
26–34, years	20.3 (19.1–21.5)	21.1 (19.8–22.5)	14.0 (11.5–16.9)	
35–49, years	31.5 (30.5–32.5)	31.8 (30.5–33.0)	27.6 (24.3–31.3)	
50–64, years	30.6 (29.1–32.2)	28.7 (27.0-30.4)	46.6 (41.8–51.4)	
**Annual family income**				< 0.001
< $20,000	14.5 (13.5–15.6)	10.4 (9.4–11.4)	45.9 (41.4–50.5)	
$20,000-$49,999	24.0 (22.6–25.4)	22.7 (21.4–24.1)	32.4 (27.4–37.7)	
$50,000-$74,999	14.1 (13.3–14.9)	14.9 (14.0-15.9)	8.3 (6.5–10.5)	
$75,000 and above	47.5 (45.7–49.2)	52.0 (50.3–53.8)	13.4 (10.4–17.2)	
**Education**				< 0.001
Less than high school	10.0 (9.2–10.8)	8.4 (7.6–9.2)	20.8 (17.8–24.3)	
High school diploma	29.2 (27.9–30.5)	27.2 (25.7–28.8)	42.7 (38.0-47.5)	
Some college	29.6 (28.3–30.8)	30.5 (29.2–31.8)	25.5 (21.3–30.2)	
College degree	31.3 (29.9–32.6)	34.0 (32.5–35.5)	11.0 (8.6–14.0)	
**Relationship status**				< 0.001
Married	45.1 (43.6–46.6)	47.3 (45.8–48.7)	29.5 (24.8–34.5)	
Never married	42.3 (40.8–43.9)	41.6 (40.1–43.1)	48.5 (43.1–53.8)	
Divorced / widowed / separated	12.6 (11.4–14.0)	11.2 (10.0-12.5)	22.1 (17.8–27.1)	
**Urbanicity**				< 0.001
Large metropolitan	55.6 (53.3–57.9)	56.3 (53.8–58.7)	50.6 (46.3–54.8)	
Small metropolitan	32.2 (30.5–34.1)	32.4 (30.4–34.4)	31.8 (27.7–36.3)	
Non-metropolitan	12.1 (10.7–13.7)	11.4 (10.0-12.9)	17.6 (13.8–22.3)	

Note. NEET = Not in employment, education, or training; NH = Non-Hispanic; CI = Confidence interval. All ps significant at the Bonferroni-corrected level of *p*<0.007

### Primary analyses

As shown in Table 2, NEET men had a distinctly different substance‑use and treatment profile than their non‑NEET counterparts. NEET men had a higher prevalence of past-year substance use treatment (4.6% vs. 1.8%; *p* = 0.008), and a higher prevalence of several substance use disorders including methamphetamine (3.5% vs. 0.8%; *p* < 0.001), prescription opioid (5.5% vs. 1.7%; *p* < 0.001) and tranquilizer/sedative use disorders (2.4% vs. 0.7%; *p* < 0.001). NEET men had a higher prevalence of opioid use disorder (heroin or prescription opioid) than non-NEET men (6.2% vs. 2.0%; *p* < 0.001). Surprisingly, NEET men had a significantly lower prevalence of alcohol use disorder compared to non-NEET men (11.9% vs. 16.3%; *p* = 0.002). The overall prevalence of any past‑year SUD did not differ significantly between groups (27.2% vs. 24.5%; *p* = 0.117).

**Table 2:** Prevalence of Substance Use Treatment, Disorders, and Recent Use Among Working‑Aged Men (18‑64 y) by NEET Status, United States, 2022 National Survey on Drug Use and Health

Substance Use Outcome	Not NEET ages 18–64 weighted % (95% CI)	NEET ages 18–64 weighted % (95% CI)	*p*-value
**Substance use treatment (past-year)**			
Any substance use treatment	1.8 (1.4–2.2)	4.6 (2.4–8.7)	0.008
**Substance use disorder (past-year)**			
Alcohol use disorder	16.3 (15.3–17.4)	11.9 (9.9–14.3)	**0.002**
Cannabis use disorder	10.2 (9.3–11.2)	12.7 (10.4–15.3)	0.025
Cocaine use disorder	0.9 (0.6–1.2)	1.3 (0.6–2.6)	0.366
Methamphetamine use disorder	0.8 (0.6–1.1)	3.5 (2.2–5.7)	**< 0.001**
Prescription opioid use disorder	1.7 (1.3–2.3)	5.5 (3.8–7.8)	**< 0.001**
Heroin use disorder	0.4 (0.3–0.7)	1.3 (0.7–2.7)	0.007
Opioid use disorder (heroin or prescription opioid)	2.0 (1.5–2.8)	6.2 (4.4–8.6)	**< 0.001**
Prescription stimulant use disorder	0.8 (0.6-1.0)	0.7 (0.3–1.6)	0.65
Tranquilizer/sedative use disorder	0.7 (0.5-1.0)	2.4 (1.7–3.5)	**< 0.001**
Any substance use disorder (drug or alcohol use disorder)	24.5 (23.2–26.0)	27.2 (24.1–30.5)	0.117
**Recent substance use (30-day)**			
Alcohol	61.3 (59.9–62.7)	34.1 (29.9–38.4)	**< 0.001**
Cannabis	20.7 (19.5–22.0)	26.6 (23.0-30.6)	0.005
Cocaine	1.4 (1.1–1.8)	1.6 (0.9–2.9)	0.649
Methamphetamine	0.7 (0.4-1.0)	3.2 (1.9–5.5)	0.005
Heroin	0.3 (0.1–0.5)	1.3 (0.6–2.7)	0.048
Fentanyl	0.1 (0.1–0.3)	0.3 (0.1–1.1)	0.311
Prescription stimulant misuse	0.8 (0.6–0.9)	0.6 (0.2–1.7)	0.672
Prescription pain reliever misuse	1.0 (0.7–1.4)	1.4 (0.9–2.4)	0.309
Tranquilizers	0.5 (0.4–0.7)	0.8 (0.3–1.7)	0.486
Sedatives	0.1 (0.0-0.2)	0.3 (0.0-1.7)	0.478
Nicotine dependence (NDSS)	10.1 (9.1–11.3)	24.4 (20.7–28.5)	**< 0.001**
**Past-month substance use frequency** ^1^			
**Cannabis (days used in past 30 days)**			0.064
1–2 days	16.6 (14.8–18.4)	9.7 (6.5–12.9)	
3–5 days	13.0 (11.1–14.9)	9.2 (6.2–12.3)	
6–19 days	19.4 (17.1–21.7)	24.6 (16.1–33.1)	
20–30 days	51.1 (48.0-54.2)	56.5 (47.2–65.8)	
**Heroin (days used in past 30 days)**			0.307
1–2 days	8.8 (2.4–15.2)	4.2 (0.9–7.4)	
3–5 days	11.2 (4.1–18.4)	8.4 (1.7–15.0)	
6–19 days	12.2 (3.5–20.9)	35.7 (9.2–62.2)	
20–30 days	67.7 (41.9–93.5)	51.8 (20.1–83.5)	
**Methamphetamine (days used in past 30 days)**			**< 0.001**
1–2 days	16.9 (6.1–27.7)	5.3 (1.1–9.4)	
3–5 days	9.4 (4.1–14.6)	6.9 (2.2–11.5)	
6–19 days	9.9 (5.1–14.6)	64.6 (41.2–88.0)	
20–30 days	63.9 (44.5–83.3)	23.3 (9.6–37.0)	
**Alcohol consumption patterns, mean (SE)**			
Days per week drank alcohol, past year	1.8 (0.04)	0.6 (0.05)	**< 0.001**
Binge alcohol frequency, past 30 days	1.5 (0.05)	0.9 (0.10)	**< 0.001**
Days had one or more drinks, past 30 days	5.3 (0.12)	2.5 (0.24)	**< 0.001**
Usual number of drinks per day, past 30 days	1.9 (0.03)	1.2 (0.11)	**< 0.001**

NEET = Not in education, employment, or training; CI = confidence interval; SUD = substance use disorder; NDSS = Nicotine Dependence Syndrome Scale. Bold = *p* < 0.0045 Bonferroni corrected. ^1^Substance use frequency variables exclude all non-users (0 days). Prescription drug use in this table refers to misuse. All estimates survey‑weighted

When looking at recent use, NEET men showed significant differences in nicotine dependence and measures of alcohol consumption but fewer differences regarding other substances. Specifically, NEET demonstrated more than twice the prevalence of nicotine dependence in the past 30 days compared to non-NEET men (10.1% vs. 24.4%, *p* < 0.001), but reported fewer binge drinking episodes (0.89 vs. 1.48 days, *p* < 0.001), fewer days drinking per week (0.61 vs. 1.81 days, *p* < 0.001), and fewer drinks per day (1.20 vs. 1.91, *p* < 0.001). Past 30-day alcohol consumption was significantly lower in NEET men (61.3% vs. 34.1%, *p* < 0.001). Frequency distributions suggest that when NEET men used methamphetamine, they had a lower prevalence of casual use (1–2 days: 5.3% vs. 16.9%) and daily use (20–30 days: 23.3% vs. 63.9%) and a higher prevalence of intermediate-frequency use (6–19 days: 64.6% vs. 9.9%; *p* < 0.001).

In our adjusted models (Table 3), with all else being equal, working-aged NEET men had lower odds of past-year alcohol‑use disorder but substantially increased odds of several SUDs. After applying the Bonferroni‑corrected α = 0.0045, NEET status was associated with lower odds of alcohol‑use disorder (aOR = 0.68, 95% CI = 0.54–0.85), and higher odds of cannabis use disorder (aOR = 1.40, 95% CI = 1.13–1.74), methamphetamine use disorder (aOR = 3.10, 95% CI = 1.82–5.28), prescription pain reliever use disorder (aOR = 2.88, 95% CI = 1.82–4.53), any opioid use disorder (heroin or prescription; aOR = 2.68, 95% CI = 1.71–4.20), and prescription tranquilizer/sedative use disorder (aOR = 3.54, 95% CI = 1.97–6.37). No significant differences emerged for cocaine, heroin, prescription‑stimulant, or any drug and alcohol use disorder, and the association with past‑year substance use treatment engagement fell below the Bonferroni-adjusted threshold for significance. Overall, the adjusted findings highlight a distinct substance use pattern in which NEET men carry disproportionate risks for high‑harm prescription and illicit drug disorders while exhibiting comparatively lower alcohol involvement.

**Table 3:** Adjusted Models Examining NEET Status as a Risk Factor for Past-Year Substance Use Outcomes Among Working-Aged Men (18-64y) in the United States, 2022

Substance Use Outcome	NEET men vs. not-NEET men (independent variable). Adjusted OR (95% CI) ^a^
Substance use treatment (past-year)	
Any substance use treatment	2.09 (1.06–4.14)
Substance use disorders (past-year)	
Alcohol use disorder	**0.68 (0.54–0.85)**
Cannabis use disorder	**1.40 (1.13–1.74)**
Cocaine use disorder	1.13 (0.49–2.59)
Methamphetamine use disorder	**3.10 (1.82–5.28)**
Prescription pain reliever use disorder	**2.88 (1.82–4.53)**
Heroin use disorder	2.23 (0.81–6.14)
Any opioid use disorder (heroin or prescription pain reliever)	**2.68 (1.71–4.20)**
Prescription stimulant use disorder	0.95 (0.39–2.32)
Prescription tranquilizer/sedative use disorder	**3.54 (1.97–6.37)**
Any substance use disorder (drug or alcohol use disorder)	1.13 (0.94–1.36)

OR = odds ratio; CI = confidence interval; SUD = substance use disorder

^a^ Adjusted for age, race/ethnicity, urbanicity, and marital status

We utilized a Bonferroni statistical correction, bolded values indicate significance at a *p* < 0.0045

## Discussion

### Principal findings

In this nationally representative study, NEET men had substantially higher odds of prescription tranquilizer/sedative use disorder, methamphetamine use disorder, and opioid use disorder compared to their counterparts. We estimated that 11.1% of working-aged men (18–64) were not engaged in employment, education or training in 2022. This is remarkable given that the U.S. enjoyed historically low rates of unemployment (less than 4%) at the time of data collection [34]. Our study adds to the literature by providing a more comprehensive picture of the NEET population that shows an overrepresentation of middle-aged and older adults [35]. Consistent with past research, we found a high prevalence of past-year SUDs among NEET men, but past 30-day use of illicit substances was similar among both groups. Importantly, NEET men showed strikingly lower rates of alcohol use and higher rates of nicotine dependence. Despite NEET men being disproportionately in rural areas where SUD treatment services tend to be in short supply, they had slightly higher prevalence of SUD treatment engagement. These findings indicate that public health interventions should be tailored by substance type (e.g., prescription drugs, methamphetamine) and designed with age- and culturally appropriate components for this vulnerable population.

Our estimate that 11.1% of men (18–64) were NEET in 2022 closely aligns with previous research indicating that 11% of prime-aged men (aged 25–54) were not participating in the labor force in 2019 [1, 2]. NEET men also had significantly higher past-year prevalence of several SUDs which is consistent with previous work that has established independent associations between NEET status and substance use [12, 24, 36].

In contrast with previous literature, NEET men had significantly lower alcohol use among several key measures including fewer binge drinking episodes, fewer drinking days per week, fewer days with any alcohol consumption in the past month, and fewer drinks per day. This finding contrasts with an earlier study using the same NSDUH dataset which found significantly higher rates of heavy alcohol use and alcohol use disorders among unemployed individuals between 2002 and 2010 [15]. However, Compton et al. defined unemployed as those without a job in the last four weeks whereas our study looked at longer-term unemployment which was defined as 52 weeks. Although alcohol consumption frequency increased in the general population during the COVID-19 pandemic [16, 37], this trend varied among different demographic groups [38, 39].

Our finding of lower alcohol use among NEET men likely reflects the income-dependent nature of alcohol consumption. Unlike most illicit substances, alcohol is considered a ‘normal good,’ meaning demand tends to rise and fall with income [40]. This behavioral economic framework is supported by pandemic-era evidence showing a polarization in drinking patterns along socioeconomic lines. A study of Swiss men demonstrated that those reporting higher COVID-19-related health concerns and/or financial anxieties reported a decrease in their alcohol consumption, while those with fewer such concerns reported an increase [41]. A large study of over 30,000 adults in the UK found a similar polarization based on socioeconomic position; individuals with low income were more likely to reduce their drinking whereas those with high income were more likely to drink more during the pandemic [42]. Given that nearly half of the NEET men in our study earned less than $20,000 annually and were over the age of 50, they may have faced heightened health and financial vulnerabilities consistent with the polarization effect which may partially explain a reduction in alcohol consumption as a protective or cost-saving response to pandemic-related stressors.

NEET men had substantially higher rates of past-year SUDs for multiple substances, but measures of past 30-day substance use were not significantly different between the two groups. This might suggest that some individuals reduced or ceased their substance use after becoming NEET due to financial constraints. This discrepancy might also indicate that comparable use of illicit substances might lead to more severe levels of functional impairment in the context of long-term unemployment, socioeconomic differences and the COVID-19 pandemic [43]. An additional explanation is a telescoping effect in which NEET men may progress more rapidly from substance use to disordered use than non-NEET men [44]. This accelerated progression may be associated with a lack of daily structure, increased psychological distress, and reduced social monitoring typically provided by employment or education. Social distancing measures, telehealth prescribing of prescription medications, lower prices for synthetic drugs and mental health co-morbidity may partially explain this specific substance use profile [18, 45–47]. For example, NEET status was shown to be associated with severe symptoms of anxiety and moderate/severe symptoms of depression and concurrent mental health and substance problems [36, 48]. Our population’s older age and over-representation of Non-Hispanic Black males may also partially explain our findings due to increased intersectional vulnerability [49, 50].

In our sample, NEET status was disproportionately concentrated among older working age adults, which contrasts with much of the existing literature that predominantly focuses on adolescent and young adult NEET populations [26, 35, 51, 52]. This pattern may reflect unique employment challenges faced by older workers during this period compared to previous economic downturns [9]. It could also be that older men have been using substances for more years, which may have increased the severity of their disorder and simultaneously entrenched their NEET status [50, 53]. The significant overrepresentation of men who identify as Non-Hispanic Black in the NEET population underscores the need for culturally tailored interventions. NEET men were significantly more likely to lack a high school diploma and far less likely to hold college degrees. These demographic patterns suggest disparities around the impact of COVID-19 on employment sectors especially among those requiring in-person work and those employing men without college credentials [54]. The concentration of NEET status among older working age adult men represents a significant shift from the traditional view of NEET as a youth-centered problem.

Although NEET men showed higher rates of treatment seeking, this difference attenuated after adjustment which suggests that interventions should prioritize enhancing treatment effectiveness and retention as well as substance-specific programs. Previous studies show mixed results, with some indicating a treatment gap among long-term unemployed individuals, while others suggest unemployment rates significantly correlate with substance use treatment admissions [55, 56]. Potential mechanisms for the attenuation include differential treatment access by rurality, demographic profile (older vs. younger), and the availability of substance specific treatment options (e.g. methamphetamines). Although our cross-sectional design precludes causal inference, several pandemic-era policy changes in SUD treatment may be relevant to understanding NEET men’s treatment patterns. NEET men in our sample were more likely to have lower incomes and may disproportionately rely on Medicaid or lack insurance which are associated with sensitivity to changes in insurance-based treatment access. NEET men were also overrepresented in non-metropolitan areas (17.6% vs. 11.4%), where policies such as telehealth expansion and relaxed buprenorphine prescribing regulations may be particularly important [57, 58].

The high prevalence of illicit substance use among the NEET population is cause for concern due to the potential for serious harms related to hospitalizations and fatal overdose especially among vulnerable populations [59]. For example, hospitalizations for stimulant use increased by 42.6% for adults aged 55–64 in California from 2017 to 2021 [60]. Beginning in 2020, over 50% of all drug overdose deaths among adults aged 25–64 are estimated to have occurred among males with at most a high school-level education [61]. During the COVID-19 pandemic, Black individuals experienced the largest percentage increase in drug overdose mortality (48.8%), surpassing overdose rates among White individuals for the first time since 1999 [62]. These findings are similar to patterns observed during the Great Recession, when prolonged unemployment among men was associated with rising deaths of despair, including drug overdoses, alcohol-related liver disease and suicides [6, 7]. These data suggest that a high proportion of NEET men in our sample are at increased risk of mortality due to their demographic and substance use profiles and likely account for a disproportionate number of overdose fatalities.

### Implications

NEET men in this 2022 nationally representative sample display unique substance use behaviors that have higher risk profiles which warrant immediate attention. Our findings highlight the urgent need for an integrated model of treatment for NEET men that addresses substance use, employment, and socio-economic barriers to workforce reentry and risk mitigation. For example, increasing educational attainment and improving economic conditions could have the potential to decrease unemployment rates and reduce substance use disorders simultaneously. Promising approaches include substance-specific programs targeting prevalent drugs among NEET men (e.g. methamphetamine, opioids), contingency management interventions that simultaneously address employment and substance use, re-education programs that target low-income communities or justice-involved individuals, and policy changes that extend health coverage for individuals transitioning out of employment to maintain treatment continuity [63]. Focusing on enhancing work-related recovery capital for NEET men engaged in treatment may have indirect benefits for SUD outcomes [64]; professional treatment programs (e.g. physician’s health programs) have high rates of success [65].

Evidence-based interventions such as overdose rescue medications and medications for opioid use disorder are critical tools for treatment and should be made easily available for low-income and uninsured populations and accessible in a wide variety of settings [66, 67]. The widespread use of synthetic drugs in the post-COVID era, and the new epidemic of stimulant and opioid co-use make these interventions critically important [68]. Early prevention strategies aimed at reducing harmful substance use among unemployed men is warranted, especially when considering that stimulant use disorders have no FDA-approved medication-based treatment options. Future studies should consider adapting substance use prevention programs tailored to unemployed youth for middle-aged and older adults of different cultural backgrounds [69]. NSDUH variables on age of first substance use and years since last employment could be used to examine whether longer substance use histories interact with NEET status among older working-age adults. Our descriptive analyses revealed elevated NEET prevalence among men identifying as sexual minorities which suggests that future research should examine the intersection of sexual minority status, NEET status, and substance use outcomes. Follow-up studies using longitudinal data are warranted to better understand any potential causal mechanisms related to the COVID-19 pandemic.

### Limitations

Our study is limited due to the self-report nature of the survey which is therefore susceptible to recall and social desirability bias. Additionally, self-report of drug class used may lead to misidentification of specific substances. Multiple testing could be a limitation, but test results (ps < 0.0045) remained significant in light of a Bonferroni correction (alpha = 0.0045). In addition, our study is limited to community-living NEET men aged 18–64 and may not be representative of certain vulnerable subpopulations such as individuals experiencing homelessness who do not use shelters or those in institutional settings. Also, the NSDUH does not include a diagnostic interview; responses to survey questions are not a direct clinical diagnosis. However, proxy diagnoses in the NSDUH are used extensively in the literature and have been validated in previous studies. While our study describes substance use outcomes among NEET men during the COVID-19 pandemic period, the cross-sectional nature of our data limits causal inference regarding pandemic-related effects. Finally, social isolation and/or loneliness may be common risk factors for both NEET status and substance use disorders. However, loneliness and social isolation constructs are not directly measured in the NSDUH. Future research should examine the potential link between social connectedness, NEET status and substance use.

## Conclusion

More than one in ten U.S. working-aged men were NEET in 2022, and they showed elevated risk of tranquilizer/sedative, methamphetamine, and prescription-opioid use disorders while showing unexpectedly lower alcohol use. These findings call for multi-level evidence-based interventions that integrate substance-specific treatments (e.g., MOUD, contingency management for stimulants) with vocational assistance, and healthcare policies that maintain insurance coverage during periods of long-term unemployment; targeted interventions should be age-appropriate and culturally tailored.

## Data Availability

The data used in this study came from publicly available datasets from the National Survey on Drug Use and Health (NSDUH). These datasets can be accessed through the Substance Abuse and Mental Health Data Archive (SAMHDA) via the Substance Abuse and Mental Health Services Administration (SAMHSA) data portal after a brief application process. Additional data from these analyses are available from the authors upon reasonable request.
